# Reasons and Risk Factors for the Initial Regimen Modification in Chinese Treatment-Naïve Patients with HIV Infection: A Retrospective Cohort Analysis

**DOI:** 10.1371/journal.pone.0133242

**Published:** 2015-07-24

**Authors:** Jianjun Sun, Li Liu, Jiayin Shen, Tangkai Qi, Zhenyan Wang, Wei Song, Renfang Zhang, Hongzhou Lu

**Affiliations:** 1 Key Laboratory of Medical Molecular Virology of MOE/MOH, Department of Infectious Disease, Shanghai Public Health Clinical Center, Fudan University, Shanghai, China; 2 Department of Infectious Disease, Huashan Hospital Affiliated to Fudan University, Shanghai, China; 3 Department of Internal Medicine, Shanghai Medical College, Fudan University, Shanghai, China; Shanghai Medical College, Fudan University, CHINA

## Abstract

**Background:**

To investigate the reasons and risk factors for modification of the first combined antiretroviral therapy (cART) currently used for HIV infected patients who were treatment naïve in Shanghai China.

**Methods:**

Making a retrospective observational research on treatment naïve patients with HIV infection who initiated cART during the period of September 1st 2005---December 1st 2013. The demographic and clinical data were collected from the first visit to the time of the first regimen modification or the last visit in December 1st, 2014. The reasons of treatment modification were recorded. Survival analysis of modification was made by Kaplan-Meier curves analysis and log rank test, and a Cox multiple regression model was constructed to identify related factors of modification.

**Results:**

A total number of the eligible participants were 3372 and 871(25.8%) patients changed their first cART regimen. The median follow up was 22 months [interquartile range (IQR) 14–39]. Among patients who modified the original regimen, drug toxicity occurred in 805(92.4%) participants and 44(5.1%) experienced treatment failure. In multiple regression analysis regimen modification was associated with patients’ age more than 40 years old (aHR 1.224, 95%CI 1.051–1.426, *P* = 0.010), CD4 less than 200(aHR 1.218, 95%CI 1.044–1.421, *P* = 0.012) and the initial regimen they received. Compared with the regimen of TDF+3TC+EFV, patients with regimen of d4T+3TC+NVP, d4T+3TC+EFV, AZT+3TC+NVP or AZT+3TC+EFV were 10.4, 8.2, 6.4, 2.5 times more likely to modify their initial regimen, respectively.

**Conclusions:**

The main reason for the regimen switch was drug toxicity and main risk factors for regimen modification were age older than 40 years, CD4 cell counts less than 200 at baseline and regimen they received. Among the 2NRTI plus 1NNRTI regimens, the co-formulation of d4T+3TC+NVP had the highest risk for modification while the regimen of TDF+3TC+EFV was the most tolerable treatment regimen in first years’ follow up.

## Introduction

There has been a rapid and successful scaling-up of combined antiretroviral therapy (cART) in China and the other parts of the world over the past decade [1–5]. Since 2003 Chinese government carry out the free cART for HIV infected patients, the mortality and morbidity of HIV infection decreased sharply in China [5]. In line with the recommendations of World Health Organization (WHO) and China HIV Council at the start of the cART, the most frequent regimens implemented in the first-line treatment comprised a dual nucleoside reverse transcriptase inhibitor (NRTI) backbone of either of zidovudine (AZT) or stavudine (d4T) plus lamivudine (3TC), with PI (protease inhibitors) or a non-nucleoside reverse transcriptase inhibitor (NNRTI): either nevirapine(NVP) or efavirenz(EFV). Because of the well-recognized metabolic toxicities, currently, d4T is not recommended as first-line regimens for treatment naïve patients [6, 7]. Since 2009, more and more HIV infected patients in China began to receive the regimen including Tenofovir disoproxil fumarate(TDF)[8] which is known to be more effective but less toxic, and many patients take 3TC and TDF as the backbone for cART[6]. Given the fact that antiretroviral drugs do not eradicate the infection, and so, at present, cART must be continued indefinitely. However, keeping the durability of these first line cART regimens is dependent on their efficacy and toxicity profile over the long term. Toxicity of cART have been associated with demographic characteristics, drug-drug interactions, comorbidities, and genetic factors [9–13], and they has been reported with all antiretroviral drugs and are one of the most common reasons for modification of treatment [14, 15]. The modification of the initial cART can do harm to the outcome of HIV infected patients [16, 17], because subsequent regimens are often more complex and expensive, and at the same time, modification of regimen requires more clinical visits and laboratory tests [18]. So it is obvious that how to choose the fittest regimen for an individual with HIV infection is extraordinarily important.

As for Shanghai in China, with the continual introduction of new antiretroviral treatment medications, such as LPV/r and TDF, the clinicians have more choice to implement the cART with more effective and less toxic medications. However there were still a number of HIV infected patients could not endure the treatment and switch the first regimen. In this paper, we evaluated the reasons for the modification of first cART in a large cohort of HIV-1 infected patients from Shanghai, China. We also analyzed the risk factors for modification. The findings of this study will provide valuable information for the first regimen selecting and visit plan making for treatment naïve patient in the future.

## Methods

### Ethics statement

This research protocols received ethical approval from the Shanghai Public Health Clinical Center Ethics Committee. The committee decided to waive the need for written informed consent from the participants studied in this analysis as the data were analyzed retrospectively and anonymously.

### Study design

For patients who initiated cART during the period of September 1st 2005—-December 1st 2013 were selected retrospectively for this survey according to the following criteria: HIV-1 positive, aged more than 16 years old, antiretroviral treatment naive, cART comprising 2 nucleoside reverse transcriptase inhibitors (NRTI) and 1 non-nucleoside reverse transcriptase inhibitor (NNRTI) or a protease inhibitor (PI). Follow up was continued until December 1st, 2014. Patients’ characteristics recorded at baseline included age, gender, marital status, HIV exposure route, initial cART regimen and CD4 cell counts. The WHO stage was assessed by the clinicians in the first visit in clinic. Antiretroviral therapy regimens were categorized as: zidovudine (AZT) and lamivudine (3TC) with either lopinavir/ritonavir (LPV/r) or efavirenz (EFV)/neviripine (NVP); d4T and 3TC with either LPV/r or EFV/NVP; tenofovir disoproxil fumarate (TDF) and 3TC with either LPV/r or NVP/EFV; and other regimen (TDF+ emtricitabine (FTC)+EFV). A small number of patients were excluded because their first regimen included the drug of IDV (indinavir), DDI(didanosine) or TMC(rilpivirine). Patients who dropped out or died in the follow-up or did not make CD4 test or WHO stage assessment were also excluded. The modification of cART regimen was defined as a change of at least one antiretroviral drug of the first regimen. For each patient, we calculated the time from the initiation of cART regimen to the switch. In the absence of these changes, follow-up ended at the last visit on December 1st 2014. The main reasons for treatment modification were classified as toxic effects, treatment failure, and other reasons(containing patient’s wish and co-infection). The treatment failure was determined by HIV load test and the criteria was in line with the WHO guideline for HIV infection treatment [7]. All of the clinical data underlying these findings are listed in the Supporting Information file **(S1 File)**.

### Analysis and Statistics

Data analysis was conducted by IBM SPSS version 19.0 (IBM SPSS, Inc., Armonk, NY, USA). Continuous variables were described using means and standard deviations or median and interquartile range (IQR) while categorical variables were described by percentages. The chi-square test was used for categorical variables and the t-test for continuous variables. We used Kaplan-Meier curves to describe the cumulative incidence of treatment modification of the most frequently used initial antiretroviral regimens, and the curves were compared by log-rank test. Risk factors of treatment modification from the time of starting cART to the last visit on December 1st 2014 were analyzed using Cox proportional hazards models. The model included: patient age at cART initiation, gender, baseline CD4 cell count, WHO stage, type of cART regimen, transmission route and marital status. All hypothesis testing was 2-sided, with a level of α = 0.05.

## Results

**Fig 1:**
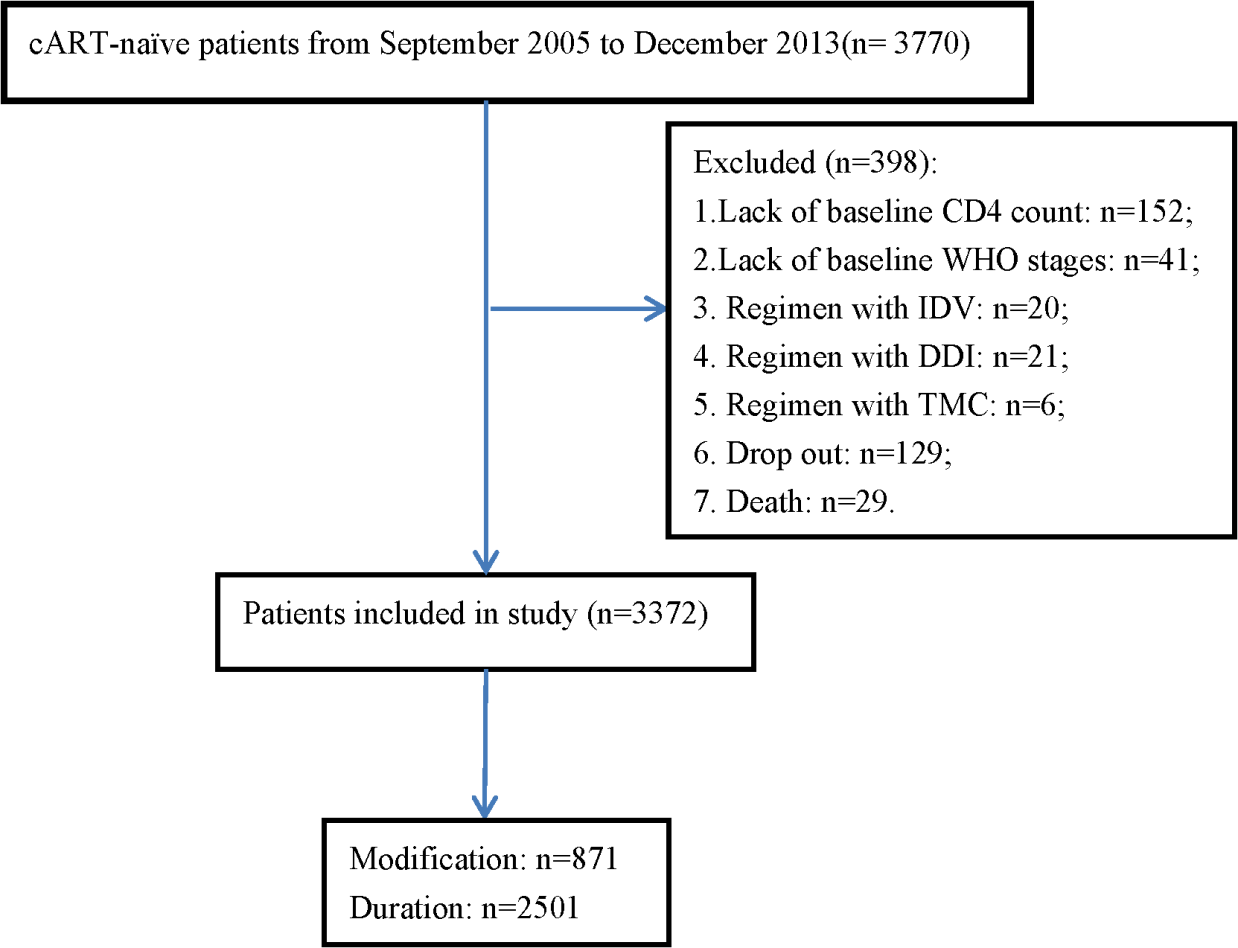
Patient selection algorithm.

Among 3770 patients who fulfilled the inclusion criteria, there were 398 cases (10.6%) excluded. So in the end, the number of participants in this analysis was 3372. See in (Fig 1). As for the regimens of cART of these patients were only two kinds, one was 2NRTI plus 1NNRTI and the other was 2NRTI plus 1PI (Given the small number of the latter regimen in China, we didn’t list them out).

From the initiation of cART to the last visit of our research, there were 871 patients switched the first regimen of cART because of drug toxicity, treatment failure and other reasons. Within the first month, 197 patients (5.8%) switched treatment; by 3 months, the number rose to 381 (11.3%), and by 12 months, to 544 (16.1%). Furthermore, the total modification rate was 25.8%. The median follow up was 22 months [interquartile range (IQR) 14–39]. Furthermore, median follow-up time for regimen of AZT+3TC+EFV was 27 months (IQR 17–42); TDF+3TC+EFV was 16 months (IQR 14–20); d4T+3TC+EFV was 24 months (IQR 13.5–40); AZT+3TC+NVP was 23 months (IQR 2–50) and d4T+3TC+NVP was 22 months (IQR 6–43.75).

**Fig 2:**
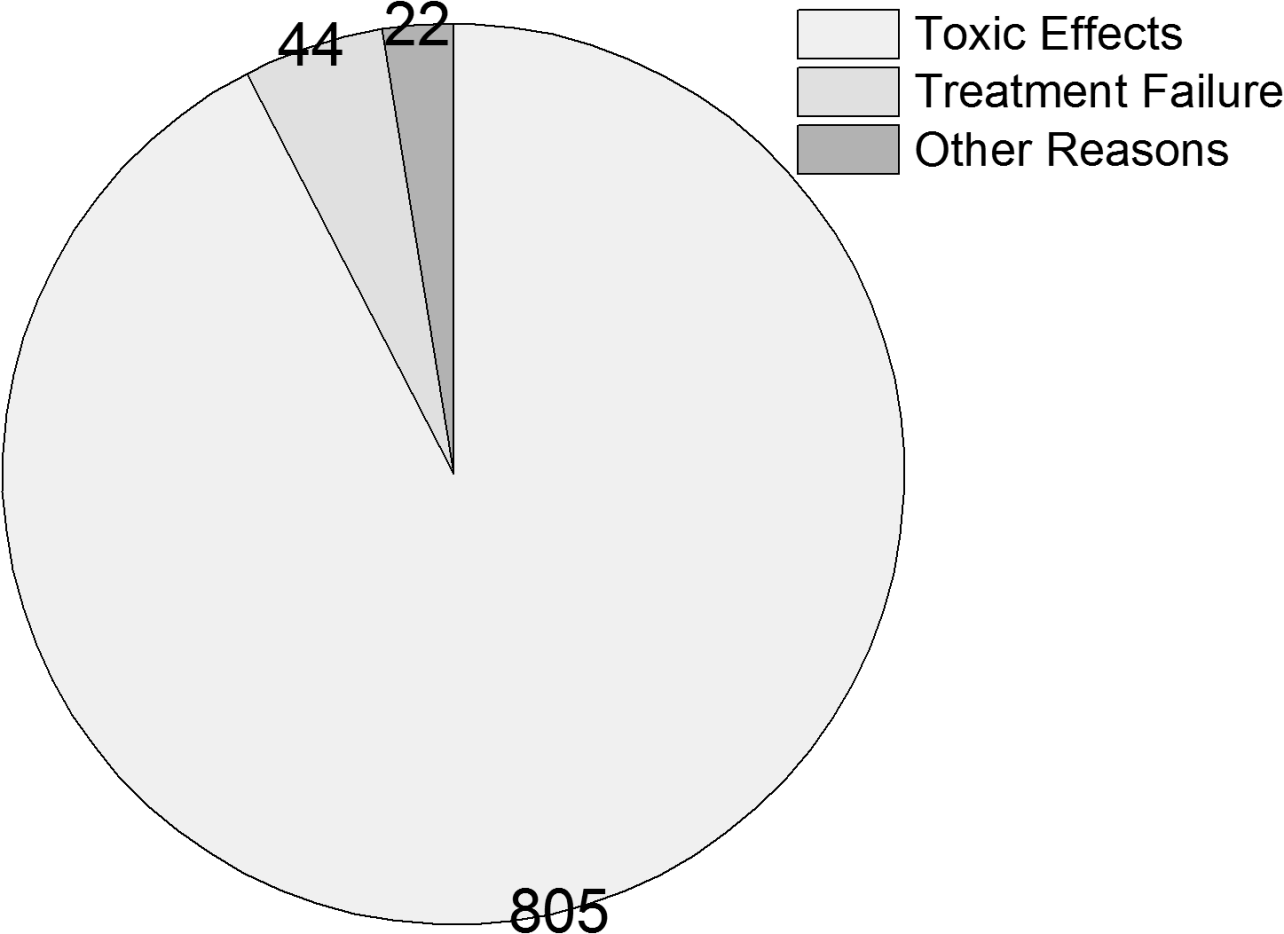
The reasons for the first regimen modification.

**Fig 3:**
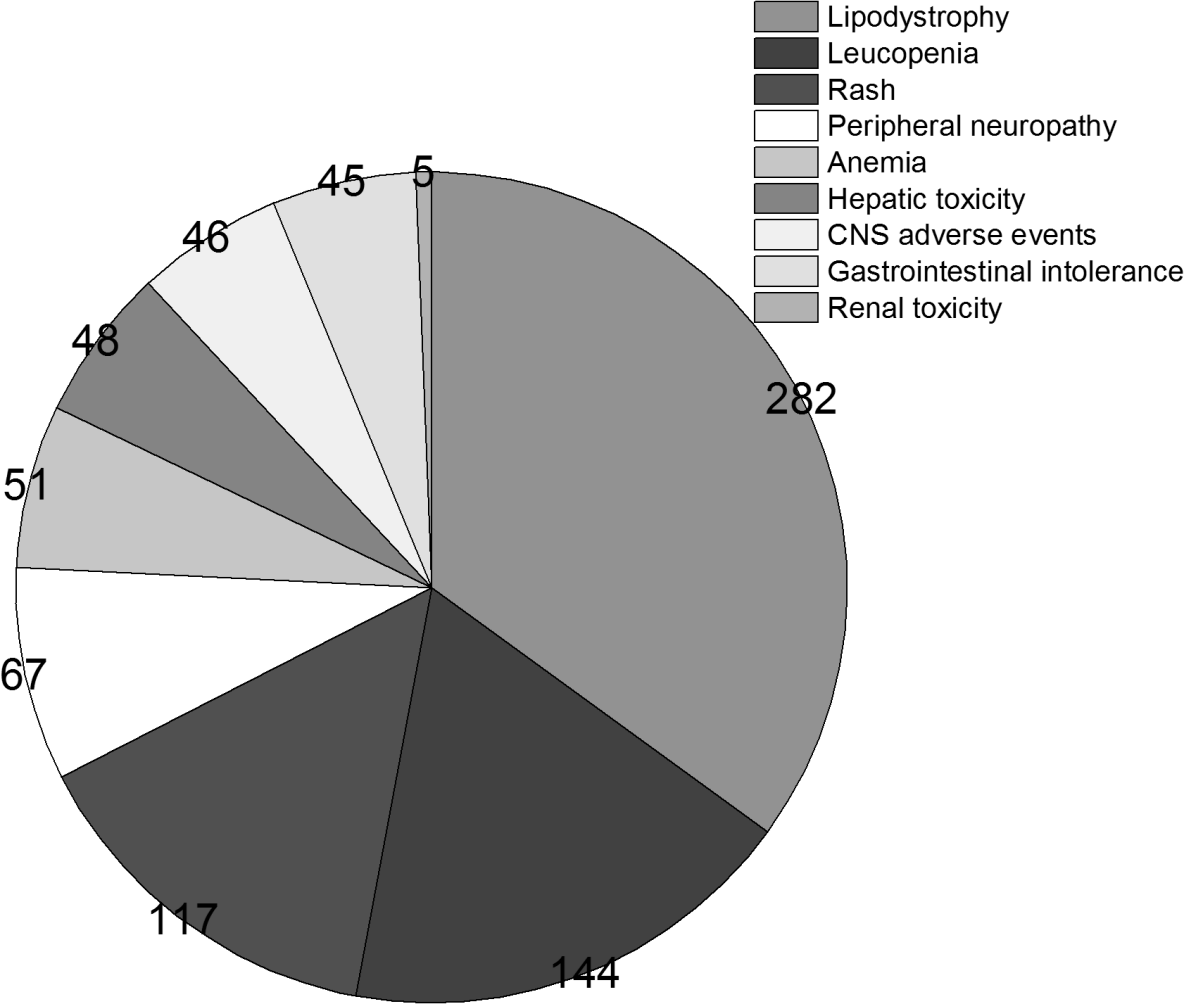
The detailed types of toxic effects.

The reasons for regimen modification were showed in (Fig 2). In detail, the other reasons of changing the regimen were patients’ wish (n = 11) and patients with HBV co-infection(n = 11). As for the 805 patients with drug toxicity, the most frequent was lipodystrophy(35.0%), followed by leucopenia(17.9%), rash(14.5%), peripheral neuropathy(8.3%), anemia(6.3%), hepatic toxicity(6.0%), CNS adverse events(5.7%), gastrointestinal tract intolerance(5.6%).They were showed in (Fig 3). The median time from initiation to treatment modification varied according to different toxic effects, ranging from 22 (IQR, 14–32) months for lipodystrophy to 2 (1–3) months for leucopenia, 1 (0.5–2) months for rash, and 9 (6–13) months for peripheral neuropathy.

We divided the participants into two groups, one was patients with regimens modification and the other was patients without modification. The difference of demographic and clinical data between these two groups were analyzed (Table 1).

**Table 1:** Patients’ characteristics and analysis of the demographics and clinical data between cART modification and none cART modification^a^.

Characteristics		Total Patients (N = 3372)	Patients with cART modification (N = 871)	Patients without cART modification (N = 2501)	*P*-value
**Age (Mean±SD)**		38.0±12.4	40.7 ±12.6	37.1 ±12.2	< 0.0001**☆**
**Gender**					< 0.0001
	Male	3034(90.0)	743(85.3)	2291(91.6)	
	Female	338(10.0)	128(14.7)	210(8.4)	
**Marital status**					< 0.0001
	Married or live together	1437(42.6)	447(51.3)	990(39.6)	
	Single, divorced, or widowed	1935 (57.4)	424(48.7)	1511(60.4)	
**Infection route**					0.0011
	Sexual route	2699(80.0)	669(76.8)	2030(81.2)	
	Non-sexual	673(20.0)	202(23.2)	471(18.8)	
**CD4 (Mean±SD)**		186.7±127.4	141.1±125.0	202.6 ±124.4	< 0.0001**☆**
**WHO stage**					< 0.0001
	Asymptomatic(stage 1&2)	282(8.4)	128(14.7)	154(6.2)	
	Symptomatic (stage 3&4)	3090(91.6)	743(85.3)	2347(93.8)	
**Type of initial cART**					< 0.0001
	2NRTI + 1NNRTI	3179(94.3)	847(97.2)	2332(93.2)	
	2NRTI +1PI	193(5.7)	24(2.8)	169(6.8)	
**Type of initial cART with 2NRTI plus 1NNRTI§**					< 0.0001
	AZT+3TC+EFV	1746(51.8)	292(33.5)	1454(58.1)	
	TDF+3TC+EFV	474(14.1)	25(2.9)	449(18.0)	
	d4T+3TC+EFV	405(12.0)	238(27.3)	167(6.7)	
	AZT+3TC+NVP	334(9.9)	141(16.2)	193(7.7)	
	d4T+3TC+NVP	207(6.1)	148(17.0)	59(2.4)	

a Unless otherwise indicated, data are expressed as number (percentage) of patients.

☆ These *P* values were calculated by t-test. The others were analyzed by chi-square test.

§ Not including the patients with regimens of TDF+3TC+NVP and TDF+FTC+EFV because few patients used these regimens.

It showed that the demographic and clinical data such as age, marital status, CD4 cell count and regimens of participants between the two groups were significantly different. In order to analyze which factors played an independent role, we implemented the multiple variants regression by Cox proportional hazards models(Table 2).

**Table 2:** Multiple regression analysis with Cox proportional hazards model for all the factors of regimen modification from the initiation of treatment to December 1st 2014.

Factors	Category	Adjusted hazard ratio(HR)	95%confidence interval(CI)	*P*-value
**Age (years old)**	>40	1.224	1.051–1.426	0.010
**Gender**	Female	1.050	0.860–1.281	0.631
**Marital status**	Single, divorced, or widowed	1.012	0.869–1.178	0.879
**Infection route**	Sexual transmission	1.051	0.891–1.239	0.558
**WHO stage**	Symptomatic (stage 3&4)	1.044	0.851–1.282	0.678
**CD4 cell count**	CD4 = < 200/ul	1.218	1.044–1.421	0.012
**Type of initial cART with 2NRTI plus 1NNRTI**	TDF+3TC+EFV	1 [Reference]		
	AZT+3TC+EFV	2.503	1.661–3.774	< 0.0001
	AZT+3TC+NVP	6.422	4.175–9.876	< 0.0001
	D4T+3TC+EFV	8.245	5.432–12.514	< 0.0001
	D4T+3TC+NVP	10.445	6.768–16.122	< 0.0001

The analysis indicated that regimen modification was associated with patients’ age more than 40 years old, CD4 less than 200 and the initial regimen they received. Compared with the regimen of TDF+3TC+EFV, patients with d4T+3TC+NVP, d4T+3TC+EFV, AZT+3TC+NVP or AZT+3TC+EFV were 10.4, 8.2, 6.4, 2.5 times more likely to modify their initial regimen, respectively.

**Fig 4:**
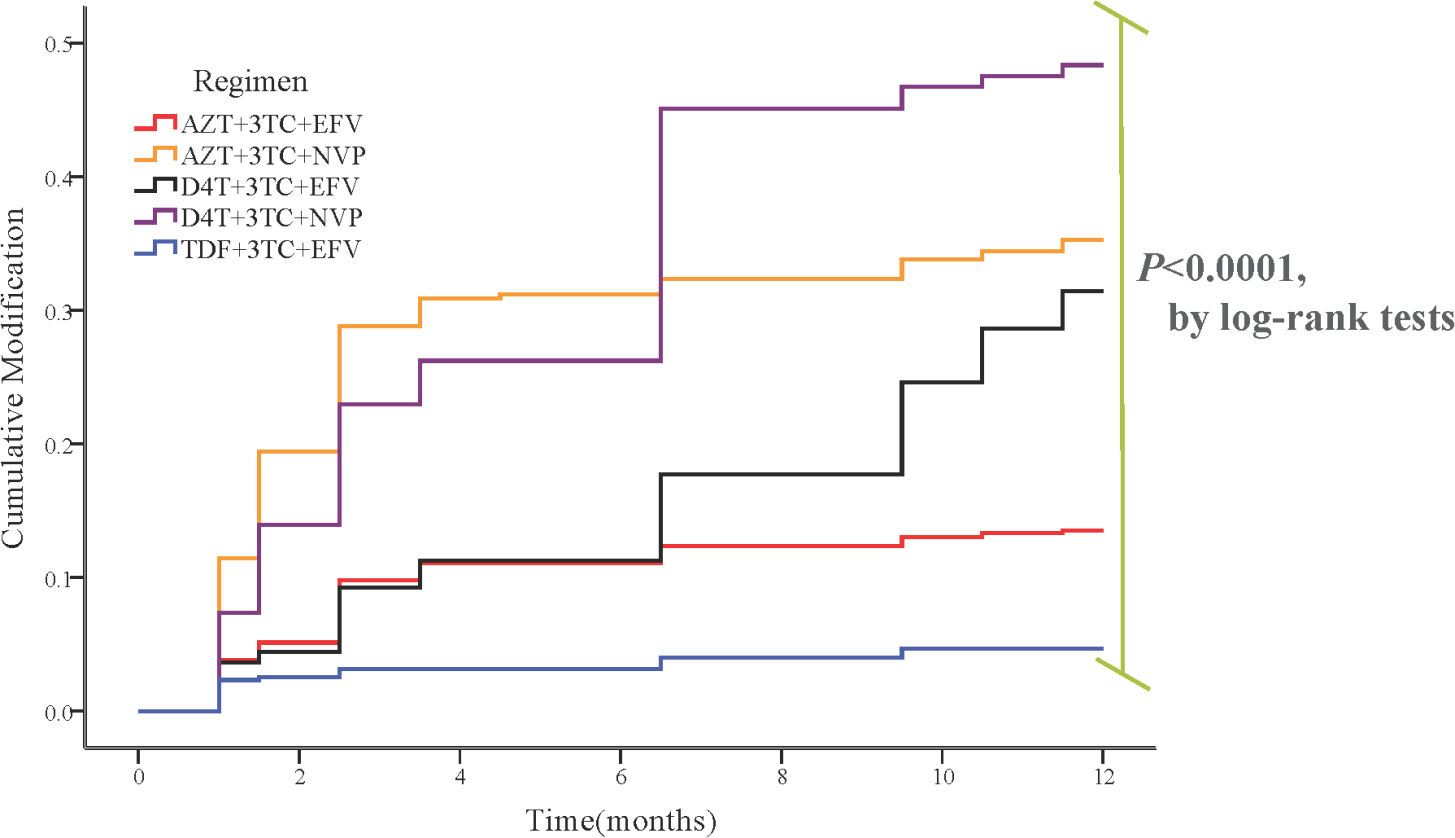
The Kaplan-Meier curve analysis for the first 12 months cART modification

Moreover, in the first year after the initiation of cART, patients with different regimen had distinct cumulative modification and this difference was significant (P < 0.0001). It can be seen in (Fig 4). Not only in the first year after initiating ART but in the whole follow-up, the regimen with the highest risk for modification was d4T +3TC+NVP and the most durable regimen was TDF+3TC+EFV.

## Discussion

In this paper, we made a retrospective analysis on the reasons and risk factors for regimen modification in China which belongs to a limited resource place for HIV treatment currently. Knowing the reasons and risk factors for the modification in China can play a positive role in optimizing the future HIV treatment. In this paper, we excluded the HIV infected patients taking indinavir, didanosine or rilpivirine as the initial regimen for cART. The reason for this exclusion is that these drugs are no longer recommended for treatment due to lack of potency and having more severe toxicities, therefore no new diagnosed patients start cART with these drugs[4].

During the follow-up from 2005 to 2014, totally 29 participants died (0.8%) and 129 patients dropped out (3.7%), this proportion is relatively small comparing with other researches[19, 20]. It was mainly because our data collected from clinic and many patients with HIV infection in China often hesitated to see a doctor and finally diagnosed in very late stage [21–23]. Patients in these situations were often admitted in our ward to receive intensive treatment for the diseases such as pneumocystis pneumonia (PCP), tuberculosis, cryptococcal meningitis, in the end, like other researches[24, 25], many of them could not survive and died in hospital finally. And these patients were not included in our clinic, so the number of patients died during the follow-up was relatively small in our research. We defined those patients who did not visit the clinic more than 6 months as losing to follow up. And in China, besides family support, the doctors and nurses spent lots of time to provide intensive therapeutic education, counseling and psychosocial support for patients because the medical resource is limited and we have little options to make when treatment failure occurs in our clinic. As the other researchers mentioned that [26], intensive therapeutic education might explains the low rate of patients drop out and treatment failure.

Among the patients who switched the first regimen, the total modification rate was 25.8% and the most common reason was drug toxicity. Furthermore, the most frequent toxic effects were lipodystrophy and peripheral neuropathy which were mostly caused by d4T[27]. D4T was formerly among the most commonly used medications in cART because of its low cost, high efficacy, and wide availability[26, 28]. However, due to the potent toxicity such as peripheral neuropathy and lipodystrophy[27, 29, 30], WHO and many countries HIV panel recommended that d4T should be phased out of cART[7, 31]. In the revised 2010 and 2013 guidelines, WHO recommended transitioning away from the use of d4T even in patients without documented virologic failure. In China, d4T was not commended for treatment naïve patients since 2009 and d4T is being replaced by other NRTIs such as TDF and AZT nowadays.

In our study, the median follow-up time for TDF+3TC+EFV was shorter than other 2NRTI plus 1NNRTI regimens. The main reason for the disparity was that TDF was introduced into China much later than other anti-HIV drugs [8]. Among all the 2NRTI plus 1NNRTI regimens in this study, AZT+3TC+EFV has the longest median follow-up time. Compared with other regimens, the drugs in this regimen were widely available and more tolerable. As for the regimen with the most risk for regimen modification during the whole follow, it was the d4T+3TC+NVP. Considering that the drug toxicity rate varied with the duration of antiretroviral therapy, we made further analysis by COX regression for the patients in the first year’s treatment. However, the regimen of d4T+3TC+NVP still had the most risk to cause treatment switch and TDF+3TC+EFV was still the most durable choice. This is consistent with the research from Njuguna C, et al[32], and TDF+3TC+EFV have already been recommended as one of the most preferred ones for HIV infected patients in resource-rich settings[6, 7]. However, TDF was also toxic and our research only enrolled patients who took this drug with short time and during the long time follow-up the bone density effects and renal toxicity should also be noticed[33].

As for the other factors which may influence the modification of the cART regimen, such as baseline CD4 cell count and patient age, we found that patients who had CD4 less than 200/ul had 1.2 times more likely to switch the first regimen than those with CD4 more than 200 and patients who were more than 40 years old had 1.2 times more likely to modified the regimen than those age below 40 years. Both of these were statistically significant. On the one hand, the WHO recommendation indicated that[7] patient’s older age or baseline CD4 cell count less than 200 was risk factor for toxic effect caused by AZT or d4T. On the other hand, patients who were much older and had lower CD4 cell count were more liable for concomitant opportunistic infection (cytomegalovirus, toxoplasmosis, pneumocystis pneumonia, or tuberculosis). As a result, more drug-drug interactions and cumulative drug toxicity would occur. Furthermore, for older patients with altered kidney function and pharmacokinetics and thus impaired drug metabolism, a higher incidence of toxic effects may occur, especially the frequent co-medication with over-the-counter drugs which may cause potential drug-drug interactions. So patients with these factors have more risk to suffer toxic effect and lead to regimen modification. However, some previous papers[34, 35] indicated that higher baseline CD4 cell counts were associated with increased rates of treatment discontinuation, frequently without switching to alternative drugs. So this contradiction was mainly because patients in their researches had lower motivation to continue an antiretroviral regimen causing adverse effects.

However, our findings should be considered in light of the study limitations. First, this study represents patients from only one center of cART and therefore, may not be generalizable to other clinics. Secondly, as a retrospective study, we can’t make up the data such as baseline HIV load, the CD4 cell count after initiating the cART. Moreover, we are unable to directly assess the outcomes of patients who dropped out and the individual adherence in every visit. However, some of the limitations reflect realities in carrying out such a study in a resource-limited setting.

## Conclusion

The main reason of modification for the first cART regimen was drug toxicity among patients without cART experience in Shanghai China. The risk factors for initial modifications were patient age older than 40, CD4 cell count less than 200 at baseline and the initial regimen they chose. Among the 2NRTI plus 1NNRTI regimens, the co-formulation of d4T+3TC+NVP had the highest risk for modification while the regimen of TDF+3TC+EFV was the most tolerable treatment regimen in first years’ follow up. With the scaling-up prescription of TDF, the related adverse effects should be monitored.

## Supporting Information

S1 File All the related clinical data. (XLSX)
